# Selectively damping materials for next-generation motion-artifact-free skin-interfaced soft bioelectronics

**DOI:** 10.1039/d5mh00700c

**Published:** 2025-06-25

**Authors:** Zehua Chen, Feng Zhang, Xiaoyan Qian, Ganggang Zhao, Zheng Yan

**Affiliations:** a Department of Chemical and Biomedical Engineering, University of Missouri Columbia MO 65211 USA yanzheng@missouri.edu; b Department of Mechanical & Aerospace Engineering, University of Missouri Columbia MO 65211 USA; c NextGen Precision Health, University of Missouri Columbia MO 65211 USA

## Abstract

Skin-interfaced bioelectronics are particularly susceptible to motion artifacts, and their increasingly miniaturized integrated circuits are mechanically fragile and prone to damage from external forces. These limitations hinder their reliability for long-term, continuous monitoring of physiological signals. Emerging selective-damping materials provide a promising route to overcome these limitations by absorbing and dissipating mechanical vibrations, thereby enhancing stability in prolonged wear. This review begins by outlining the challenges that motion artifacts pose for soft bioelectronic devices and the current mitigation strategies, followed by an introduction of emerging damping material design approaches tailored to the requirements of skin-interfaced bioelectronics. It further highlights the application of selective-damping materials in soft bioelectronics, with an emphasis on biosensing (electrophysiological and electrochemical signals) and mechanical shock protection. Lastly, several challenges that need to be addressed are discussed before the practical deployment of soft bioelectronics integrated with selective-damping materials.

## Abstract 2

Selective-damping materials in skin-interfaced bioelectronics can absorb and dissipate vibrations, reducing motion artifacts and enhancing stability. This review highlights biosensing and shock-absorption applications and current challenges.

## Graphical abstract

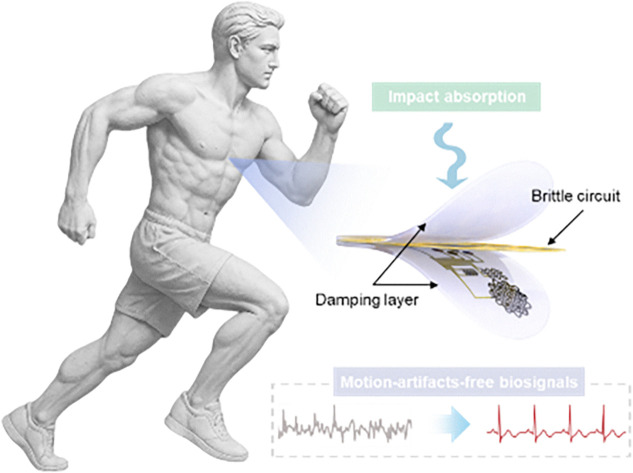

**Wider impact**

Selective-damping materials are poised to redefine how we design and deploy skin-interfaced bioelectronics by minimizing motion artifacts and protecting fragile components. This review highlights recent progress in selectively damping materials for targeted low-frequency filtering, including viscoelastic polymers, hydrogels, and acoustic metamaterials. These developments hold substantial interest in healthcare and beyond because long-term continuous sensing becomes increasingly important to disease management, and robust devices that provide continuous, artifact-free signals over prolonged periods that can greatly improve both clinical decision-making and patient quality of life. Moreover, these strategies of designing damping materials can be adapted to different application scenarios, reflecting a general trend toward more adaptive smart materials. By detailing design fundamentals and emerging case studies, our review underscores the necessity of seamlessly integrating selective-damping materials into skin-interfaced bioelectronics. We anticipate that building on these foundational insights will accelerate the arrival of next-generation devices, enabling a future in which wearable, multifunctional bioelectronics operate with unprecedented reliability in real-world conditions.

## 1. Introduction

Recent advances in materials science and flexible electronics have enabled the seamless integration of bioelectronic devices onto the skin surface, enabling a direct interface between daily physiological monitoring and clinical diagnostics.^1–3^ By conforming to the body's soft, curvilinear structures, these systems can collect vital health parameters during daily activities with minimal discomfort. Beyond comfort and wearability, these skin-integrated systems offer multifunctional sensing capabilities, capturing a diverse range of physiological signals: biomechanical (*e.g.*, stress, pressure, and deformation), electrophysiological (*e.g.*, electrocardiogram and electromyogram), and electrochemical (*e.g.*, sweat biomarkers) signals.^4^ Skin-interfaced soft bioelectronics hold great potential to transform traditional healthcare models by enabling continuous human health data acquisition and active physiological intervention, thereby enhancing disease understanding and facilitating targeted treatments.^5–8^

**Fig. 1:**
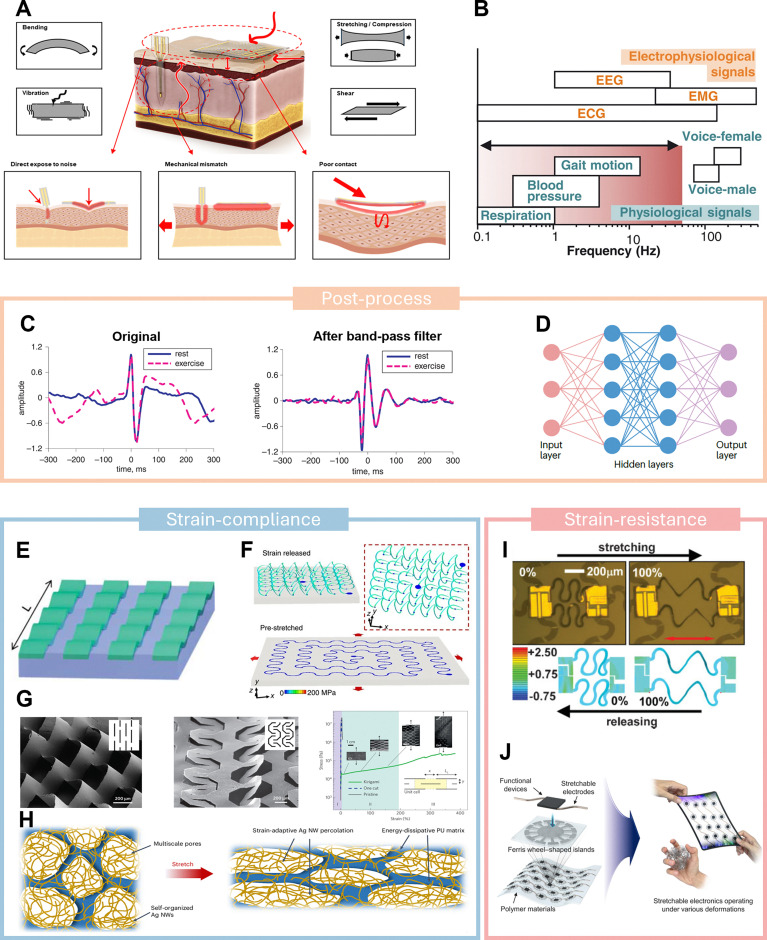
Motion artifacts and current strategies. (A) Mechanical factors contributing to noise from motion artifacts and types of mechanical stimulations in human bioelectronics applications. Reproduced with permission.^15^ Copyright 2024, American Chemical Society. (B) Representative frequency ranges of human mechanical (blue) and electrophysiological (orange) biosignals at 27° to 45 °C. Reproduced with permission.^13^ Copyright 2022, American Association for the Advancement of Science. (C) Traditional band-pass filtering method for processing ECG signals under rest and exercise conditions. Reproduced with permission.^21^ Copyright 2020, The Institution of Engineering and Technology. (D) Signal processing method based on machine learning. Reproduced with permission.^4^ Copyright 2024, Springer Nature Limited. (E) Wavy, stretchable single-crystal Si devices built on an elastic substrate. Reproduced with permission.^22^ Copyright 2006, American Association for the Advancement of Science. (F) 2D serpentine structure bonded at stretched soft elastomeric substrate. Reproduced with permission.^23^ Copyright 2017, The Author(s). (G) Examples of microscale Kirigami patterns. Reproduced with permission.^24^ Copyright 2015, Springer Nature Limited. (H) Scheme illustration of the strain-insensitive porous silver nanowire nanocomposites elastomer in original (left) and stretched (right) states. Reproduced with permission.^25^ Copyright 2024, Spring Nature. (I) Optical microscopy images and FEM-derived distributions of the stretchable silicon integrated circuits with non-coplanar bridging interconnects. Reproduced with permission.^26^ Copyright 2009, Wiley-VCH Verlag GmbH & Co. KGaA, Weinheim. (J) Schematic illustration of stretchable electronics with the Ferris wheel-shaped island (FWI) array in Ecoflex. Reproduced with permission.^27^ Copyright 2022, The Authors.

To fulfill this potential, soft bioelectronics must maintain continuous and stable high-quality signal collection without disrupting normal daily activities. However, achieving these requirements poses a daunting challenge. One of the primary barriers to long-term, real-world applications of skin-interfaced soft bioelectronics is the prevalence of motion artifacts.^4^ Compared to implantable devices, skin-mounted systems are highly exposed to external forces and mechanical stress, and are particularly vulnerable to severe artifact signals. These devices are subject to both internal mechanical stress and external environmental forces, making them more susceptible to signal disruption.^9^ Specifically, motion artifacts from the body typically arise at the device–skin interface, where activities such as bending, stretching, vibration, and shear stress induce mechanical strain,^10,11^ as illustrated in Fig. 1A. This strain can alter the electrical properties of both the bioelectronics and the interface, leading to signal distortion. For electrophysiological signals (such as electrocardiogram (ECG), electroencephalogram (EEG), electromyogram (EMG), *etc.*), both active body motions (walking, running, and jumping) and passive body activities (breathing, heartbeat, and pulse) contribute significantly to motion artifacts,^4,12,13^ as shown in Fig. 1B. While clinical settings often require patients to remain still to minimize artifacts from active motions, this strategy is ineffective against passive mechanical noise.^4,14,15^ On the other hand, environmental factors such as impact, friction and vibration in real life can also cause more complex interference to biosignals. Current devices often include delicate but fragile electronic components, which are vulnerable to shocks, impacts, or low-frequency mechanical vibrations, leading to large noise or malfunctions.^9,13^ These external forces can either directly damage the fragile electronic components or transfer mechanical energy to the device–skin interface.^16,17^ As a result, ensuring the stability of these soft bioelectronic systems becomes increasingly difficult in dynamic conditions. Another critical challenge is accurately distinguishing target biological signals from motion artifacts, as dynamic noise from mechanical stimuli is often embedded within recorded signals.^18,19^ This overlap makes it difficult to filter out noise while preserving the integrity of the desired physiological signals. Moreover, the complexity of these mechanical interferences increases the difficulty of developing effective noise-reduction strategies, especially when multiple signals are being recorded simultaneously.^20^

To date, many efforts have been made to eliminate motion artifacts in biosignals. The most common approach is post-processing, which employs signal processing techniques such as band-pass filtering to remove noise from raw signals^28–32^ (Fig. 1C). This approach has successfully reduced noise in various physiological signals, including electrophysiological signals,^4,15^ pulse,^33^ and respiration.^34^ Furthermore, with the rapid development of artificial intelligence technologies in recent years, machine learning and deep learning models have also emerged as powerful tools for mitigating signal noise^35^ (Fig. 1D). However, for complex dynamic noise, such signal post-processing methods are less effective.^36,37^ Moreover, post-processing inevitably results in signal distortion or information loss.^20,38^ Due to the limitations of post-processing techniques, attention has shifted to device-level design strategies to proactively mitigate the impact of mechanical stress. A frequently adopted strategy is “strain-compliance,” which aims to reduce the mechanical energy transferred to the device by lowering its effective modulus. Representative device structure designs, such as wavy geometries,^22^ serpentine interconnects,^23^ and Kirigami architectures^24^ (Fig. 1E–G), diffuse mechanical energy by allowing controlled deformation of noncritical regions and reduce the modulus, enabling stretchability in intrinsically non-stretchable materials.^39–41^ Thin, compliant substrates can also conform to skin motions without producing large stress gradients, thereby minimizing the risk of partial delamination. Additionally, emerging conductive elastomeric composites show remarkable potential for mitigating mechanical disturbances in dynamic conditions, due to their ability to maintain high conductivity under frequent deformation. A conductive phase-separated porous silver nanowires (AgNWs) nanocomposite that is insensitive to mechanical strain has been developed, as displayed in Fig. 1H, enabling stable biosignal recording under dynamic scenarios.^25^

Another strategy, referred to as “strain-resistance,” strategically increases the effective modulus in select regions to shield sensitive components from mechanical deformation and energy transfer. In this design, the device includes high-stiffness “islands” or layers to protect critical electronic components (*e.g.*, transistors or electrodes), while the surrounding substrate or “bridge” regions employ softer, lower-modulus materials. Such a contrast in stiffness ensures that most external strain is absorbed by the more compliant regions, minimizing dimensional changes in high-modulus islands. Consequently, delicate electronic components remain structurally and electrically stable under stretching or bending motions. The strain-resistance strategy can be implemented in multiple forms. One widely adopted form is the “island-bridge” geometry,^26^ where rigid, small-footprint device islands are connected through serpentine or wavy interconnects, as shown in Fig. 1I. When tension is applied, these bridging sections deform to accommodate strain, so the stiff islands remain mostly strain-free. Another method involves placing high-modulus layers beneath critical device regions,^27^ creating a localized barrier against mechanical distortion, as Fig. 1J illustrated. These high-modulus islands or layers are essentially “strain-resistant,” allowing sensitive electronics on them to experience less mechanical stress during motion. This reduced deformation in turn protects the electronic performance from large fluctuations or noise spikes associated with movement. At the same time, the surrounding flexible regions preserve the overall conformity and comfort needed for skin-interfaced or implantable devices, preventing excessive strain at the user–device interface.

Additional strategies focus on improving skin adhesion by employing specialized adhesives or micro-engineered surfaces, such as pillar arrays or suction-cup-inspired geometries, to enhance contact and minimize lateral shifts.^4,42^ These approaches help stabilize the electrode–skin interface, reducing both baseline drift and random spikes. Furthermore, mechanical and adhesive refinements can be coupled with sophisticated signal-processing techniques. For example, a multi-channel layout can be employed in which one channel records the primary biosignal while another tracks motion-induced disturbances; real-time subtraction of the noise channel yields a cleaner, artifact-free waveform.^35^

An emerging strategy involves the use of damping materials that intrinsically absorb or dissipate mechanical energy, effectively reducing motion artifacts. Damping refers to the reduction of unwanted mechanical vibrations through the dissipation of the associated mechanical energy as heat during impacts.^43^ Common soft damping materials, such as biological tissues,^44–46^ viscoelastic polymers,^47–50^ and meta-materials,^51^ mainly exploit the high friction among molecular chains^52^ and/or the cyclic breaking and recovery of viscous weak bonds^13^ to efficiently dissipate mechanical energy. Specifically, optimal damping performance is achieved when the relaxation time of the damping material matches the frequency of the absorbed mechanical vibration, a relationship quantified by the Deborah number (De).^13,52–54^ The Deborah number is a dimensionless parameter that relates an intrinsic relaxation time (*τ*_r_) of material to the characteristic time scale of an applied deformation or vibration (*τ*_t_). It can be expressed as:
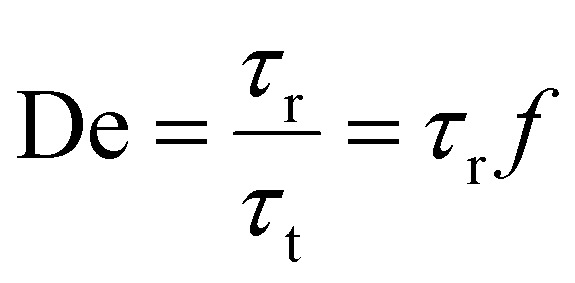
where *f* = 1/*τ*_t_ is the vibration frequency. For example, when De ≈ 1, the relaxation time matches the vibration period, maximizing viscoelastic energy loss and therefore damping efficiency, while the energy dissipation performance of the material is poor when De is much larger or much smaller than 1. This allows for the selective filtering of vibration noise at specific frequencies through rational material design. Once the primary sources of motion artifacts and their typical frequency distribution are identified, researchers can design damping materials specifically for the targeted noise spectrum. By aligning the material's relaxation time with the frequency band of the noise, vibrations in those specific frequencies can be effectively attenuated. Through this integrated approach, combining noise-source analysis with strategic material optimization, it becomes possible to customize materials to achieve highly efficient energy dissipation at the most common motion-artifact frequencies. These tailored damping materials are termed “selective damping materials”, where “selective” means the capacity to preferentially dissipate mechanical energy within a targeted frequency band while allowing higher-frequency physiological signals to pass with negligible attenuation.^13,15,52^ This strategy significantly suppresses motion artifacts and thereby provides a promising pathway toward the long-term, stable operation of soft bioelectronics.

**Fig. 2:**
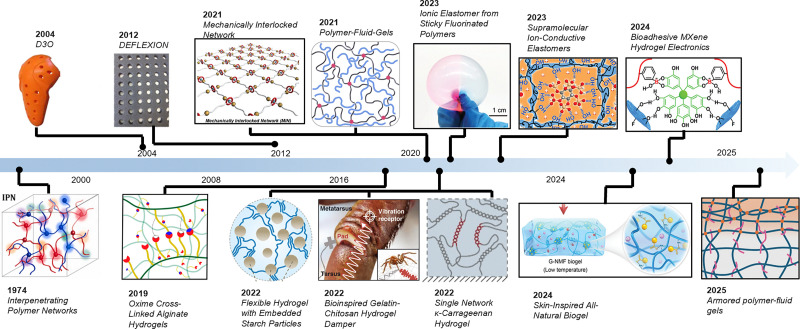
Development path of damping materials. Reproduced with permission.^13,52,55–58,60,65–70^ Copyright 2017, 2019, 2022, American Chemical Society. Copyright 2021, 2023, 2024, Wiley-VCH GmbH. Copyright 2021, Spring Nature. Copyright 2020, 2023, Elsevier. Copyright 2022, 2025, American Association for the Advancement of Science.

This unique capability of damping materials has enabled diverse applications in soft bioelectronics, such as biosensing with mitigated motion artifacts, shock absorption, and others.^52,55–57^ Numerous studies have explored the incorporation of damping materials into soft bioelectronics, leading to significant advancements in functionality and reliability. Fig. 2 illustrates the developmental trajectory of damping materials in recent years, demonstrating their growing importance in soft bioelectronics and related biomedical fields. Early efforts primarily focused on elastomeric matrices with basic viscoelastic properties, aiming to absorb mechanical energy and mitigate low-amplitude vibrations. Subsequently, researchers integrated advanced chemistries, such as interpenetrating polymer networks and dynamic supramolecular assemblies, to optimize energy dissipation and enhance material resilience under cyclic loading. Innovations in hydrogel-based damping systems further accelerated this progress by enabling precise tunability of mechanical properties through reversible cross-linking mechanisms and solvent-dependent swelling behaviors. By tuning the mechanical and chemical properties of damping materials, researchers can create systems that selectively attenuate specific mechanical noise sources, significantly improving the precision of biosignal monitoring devices.^13,58^ These materials have also proven effective in shock absorption^52,59,60^ and other areas, including self-healing skin^61^ and energy storage,^62–64^ further expanding the scope of their application in wearable technologies. These advancements are driven by increasing clinical and industrial demands, particularly the need to suppress motion-induced noise in wearable bioelectronics and to extend device longevity under continuous mechanical stress. Today's damping materials not only demonstrate superior strain tolerance and mechanical stability but also accommodate evolving design criteria, including biocompatibility, degradability, and multi-functional responsiveness.

To contextualize the role of selective-damping materials within the broader landscape of motion-artifact mitigation, Table 1 contrasts SDM-based solutions with the other strategies, including post-processing, strain-compliance designs, strain-resistance layouts, high-adhesion interfaces, and strain-insensitive composites, thereby clarifying where SDMs provide distinctive advantages and where other strategies remain complementary.

**Table 1:** Comparison of different noise reduction strategies

Strategy	Examples	Mechanism	Durability	Reported SNR gain	Artifact suppression band	Typical fabrication methods	Ref.
Post-processing filters	Band-pass, CNN/LSTM filters	Digital removal of noise frequencies; data-driven decomposition	Unlimited (software)	<20 dB	Tunable (software defined)	Firmware update or cloud pipeline	28–37
Strain compliance	Wavy films, serpentine interconnect, Kirigami meshes	Lower effective modulus so skin motion is accommodated by stretchable paths	Usually >1000 bend cycles	—	Broad	Photolithography/laser cutting	22–24,39–41
Strain resistance	Island-bridge geometries, stiff underlayers	Localize strain in compliant bridges; keep active “islands” rigid	Usually >1000 bend cycles	Usually <20 dB	Broad	Multilayer lamination	26,27
High-adhesion interfaces	Micropillar & suction-cup skins	Minimize lateral slip at electrode–skin interface	Some adhesive fatigue in 1–3 days	<10 dB	Broad	Micro-molding or laser texturing	4,42
Strain-insensitive composites	AgNW phase-separated elastomer	Maintain conductivity under strain	>10^5^ strain cycles	—	Broad	Solution casting	25
Selective-damping materials (SDMs)	κ-Carrageenan hydrogel, gelatin–chitosan damper, PTFEA-*co*-PFOEA ion-elastomer, MXene-hydrogel	Frequency-matched energy dissipation	Hydrogels: 24–72 h without humectant; ion-elastomers: >10^5^ cycles	20–50 dB	Targeted, usually less than 60 Hz	Casting, coating	13,52,55–58,60,65–70

In this review, we summarize the potential, progress, and challenges of soft bioelectronics integrated with selective-damping materials (SDM). Firstly, we provide an overview of the internal energy dissipation mechanisms in damping materials. Based on this, we discuss targeted SDM material designs aimed at eliminating motion artifacts, which are essential for developing high-precision, artifact-free bioelectronics. Subsequently, we highlight recent advances in the applications of SDM in soft bioelectronics, such as biosensing with mitigated motion artifacts, shock absorption, and others. Finally, we discuss future opportunities and potential challenges in the broader adoption of these intriguing materials in soft bioelectronics, emphasizing the promising avenues for further research and development. We believe that the strategic integration of SDM into soft bioelectronics holds transformative potential, which can significantly enhance device performance, reliability, and functionality, paving the way for next-generation soft bioelectronics that are effective and sustainable.

## 2. Mechanism of damping materials

**Fig. 3:**
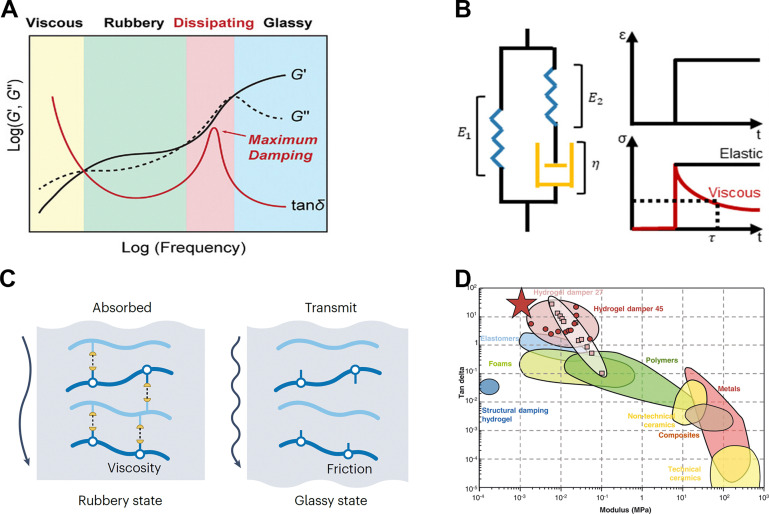
Damping mechanism of viscoelastic materials. (A) Typical rheology of viscoelastic polymers. Reproduced with permission.^52^ Copyright 2023, Wiley-VCH GmbH. (B) A basic viscoelastic model represented by the Maxwell model. Reproduced with permission.^15^ Copyright 2024, American Chemical Society. (C) Schematic illustration of the working mechanism for a damping device with band-pass filtering functionality. Reproduced with permission.^4^ Copyright 2024, Springer Nature Limited. (D) Comparison graph of modulus and loss factor values for various material types. Reproduced with permission.^13^ Copyright 2022, American Association for the Advancement of Science.

As shown in Fig. 3A, viscoelastic damping materials exhibit a glass transition at specific frequencies/temperatures, which significantly influences their damping behavior.^52,71^ Below the glass transition, polymer chains exhibit high mobility and interact through transient, weak interactions, generating a “rubbery” response with high viscous losses and, consequently, significant damping. As the frequency (or temperature) rises above this transition region, the polymer network becomes stiffer and less capable of dissipating energy, shifting into a “glassy” regime with substantially reduced damping performance. Fig. 3B illustrates a commonly used viscoelastic mechanical model (for example, the standard linear solid), comprising elastic spring elements (*E*_1_, *E*_2_) and a dashpot (*η*).^72^ Under an applied stress, the elastic components store deformation energy, whereas the dashpot (viscous) element dissipates that energy over time. This time- or frequency-dependent interplay between the spring and dashpot underlies viscoelastic damping, enabling the material to respond elastically at short timescales yet gradually relax stress through viscous flow at longer timescales. Within the rubbery state, the materials function with a viscous damping mechanism, effectively dissipating mechanical energy, while the modulus of the material increases above the transition frequency, leading to a reduction in its damping performance of mechanical vibrations,^13,73,74^ as illustrated in Fig. 3C. This characteristic makes viscoelastic polymers especially suitable for applications requiring the absorption of low-frequency mechanical noise, such as the filtering of motion-artifacts from physical,^52^ electrophysiological,^13,58^ and electrochemical^75^ sensors. By adjusting or broadening the glass transition range of elastomers through various material design strategies, it is possible to target and filter specific frequency bands selectively.

Different types of damping materials exhibit varying damping performances (Fig. 3D), which can be quantified by the loss factor (tan *δ*), defined as the ratio of the loss modulus (*G*′′) to the storage modulus (*G*′).^53,65^ Generally, a higher tan *δ* value indicates superior damping performance. Common natural damping materials such as adipose^76^ and rubber^43^ typically exhibit low tan *δ* values. While these materials offer basic damping properties, they often fall short of the requirements for more demanding applications. In contrast, emerging hydrogel-based dampers often combine moderate to low modulus with potentially large tan *δ* values.^13^ Hydrogel-based damping materials can dissipate energy through the reversible breaking and reformation of weak interactions or bonds. Under external force, these weak bonds within the hydrogel network break and then re-form, a dynamic process that consumes substantial energy. As a result, vibrations or noise at specific frequencies or intensities are effectively dissipated or filtered. Likewise, elastomer-based damping materials leverage their viscoelastic nature to absorb mechanical disturbances across a tunable range of frequencies. Additionally, compared with hydrogel-based damping materials, elastomer-based damping materials typically offer superior mechanical properties and are not prone to the common drying issues that often affect hydrogel systems. These materials will be discussed in detail in the next section.

## 3. Material design of damping materials

The starting point for selecting or designing an SDM strategy is usually to define the operating window, which includes: (1) a damping dissipation window that coincides with the dominant motion artifact frequency, and (2) a signal transmission band in which damping must be minimized so that the target biosignal can be transmitted without attenuation.^13,52,58^ Motion artifacts resulting from human activities predominantly occur within the low-frequency range (0.01–50 Hz),^13,77^ encompassing daily activities such as breathing (0.1 to 1 Hz), heartbeats (0.3 to nearly 4 Hz), gait movements (1 to 15 Hz), and strenuous exercise (15–50 Hz). Consequently, damping materials used in soft bioelectronics should exhibit outstanding damping performance across this frequency spectrum. In contrast, the information-bearing portions of most electrophysiological waveforms lie above 80–100 Hz (such as ECG, EMG, and EEG), whereas electrochemical and biomechanical outputs are quasi-DC and therefore insensitive to the high-frequency band of the damping curve. Therefore, when the application scenario involves electrophysiological signal monitoring, it is necessary to ensure that the loss factor of the damping material is at a low level in this frequency band. Based on this framework, the material properties of SDM materials for skin-interface bioelectronics are obtained: high loss factor (tan *δ* ≥ 0.5) throughout 0.01–50 Hz, followed by a rapid drop (tan *δ* ≤ 0.1) beyond ∼80 Hz to preserve electrophysiological fidelity. For SDMs selection of electrochemical and biomechanical signals, material selection generally needs to focus only on damping performance in the low-frequency range. Plotting tan *δ* – frequency curve obtained from dynamic mechanical analysis can reveal whether a candidate material satisfies the criterion. For the design of motion-artifacts-free bioelectronics, the selected damping material should exhibit a high tan *δ* value within the damping dissipation window.^4,78^

Designing damping materials essentially relies on structural modifications that manipulate polymer chain flexibility and intermolecular forces to tune their glass transition range.^4,15^ Different types of SDMs rely on diverse damping mechanisms. In the following sub-sections, we present representative SDM designs for each material category and elucidate their specific damping mechanisms.

### 3.1. Elastomer-based damping materials

**Fig. 4:**
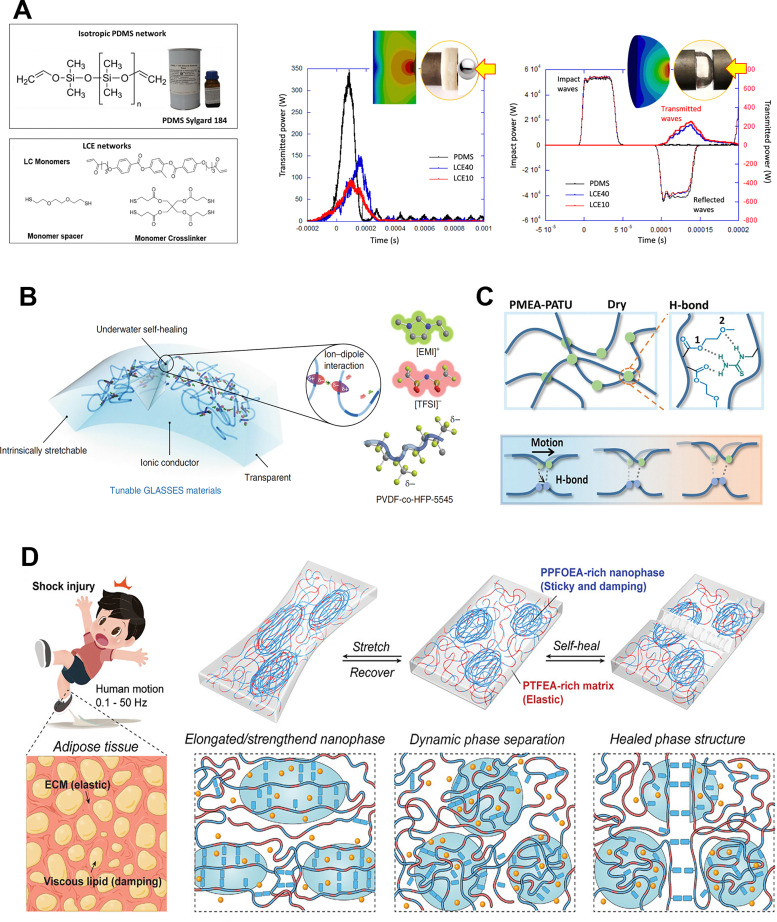
Design of damping materials based on elastomer. (A) Chemical structure of a nematic liquid crystal elastomers (LCEs) and deformation field distribution in the elastomer. Reproduced with permission.^79^ Copyright 2021, The author(s). (B) Design of a gel-like, aquatic, stretchable, and self-healing electronic skin. Reproduced with permission.^61^ Copyright 2019, Spring Nature. (C) Schematic for the synthesis of PMEA-PATU by radical copolymerization, the hydrogen bond interaction between MEA and ATU units in dry state, and the weak H-bond from low temperature to high temperature. Reproduced with permission.^80^ Copyright 2021, Wiley-VCH GmbH. (D) Highly damping and self-healing ionic elastomer derived from the dynamic phase separation of adhesive fluorinated polymers. Reproduced with permission.^52^ Copyright 2023, Wiley-VCH GmbH.

Elastomers typically exhibit outstanding mechanical properties and durability, making them highly promising materials for skin-interface bioelectronics. Elastomer-based damping materials can be engineered by incorporating reversible crosslinks, chain reorientation, or phase transitions into the polymer backbone or network, while also leveraging multiphase structures, filler composites, or glass-transition temperature control to achieve a balance between high elasticity, reversible deformation, and enhanced damping performance. For example, a recent study developed nematic liquid crystal elastomers (LCEs), as shown in Fig. 4A, which are soft, rubbery materials incorporating liquid-crystalline (LC) ordering into their polymeric network.^79^ Unlike ordinary rubbers, LCEs exhibit a stress–strain plateau caused by the rotation or reorientation of the LC director under deformation, enabling large strains at relatively low stress. Their internal nematic ordering can also rearrange under mechanical force, leading to high damping and slow stress relaxation. LCEs show high loss factor tan *δ* and remain highly dissipative through frequencies from roughly 0.1 Hz to tens of kHz. Another approach involves blending an amorphous polymer with an ion-containing compound that is chemically compatible. Although some mechanical performance is sacrificed compared to the original viscous polymer, the resulting composite can still exhibit viscoelastic properties. For instance, introducing the ionic liquid 1-ethyl-3-methylimidazolium bis(trifluoromethylsulfonyl)imide ([EMI][TFSI]) into the fluorinated elastomer poly(vinylidene fluoride-*co*-hexafluoropropylene) (P(VDF-HFP)) facilitates ion–dipole interactions and amplifies hydrogen bonding as well as other dynamic linkages^61^ (Fig. 4B). The synthesized composite proves to be tough, stretchable, and self-healing. Recently, advancements have been made in developing elastomers with high tan *δ* values, suitable for a broader range of conditions. For example, a pressure-sensitive adhesives (PSAs),^80^ shown in Fig. 4C, synthesized through the copolymerization of 2-methoxyethyl acrylate (MEA) and *N*-allylthiourea (ATU), has demonstrated the ability to maintain a tan *δ* value close to 1 across a wide temperature range. Additionally, a phase-separated fluorinated ionic elastomer comprising short-chain (TFEA) and long-chain (PFOEA) monomers, along with LiTFSI, is designed to mimic the structure of human fat, and demonstrates a loss factor exceeding 1 at typical human-motion frequencies (0.1–50 Hz), while maintaining 2000% elongation and self-healability (Fig. 4D).^52^

### 3.2. Hydrogel-based damping materials

Hydrogel-based damping materials, including tough hydrogels or supramolecular gels with reversible cross-links can dissipate mechanical stress. These often exhibit strain stiffening or shear thickening, specifically attenuating low-frequency mechanical noise while transmitting stable signals. At the critical gel point, gel systems exhibit viscoelastic properties (tan *δ* ≈ 1) across a wide frequency range.^81^ However, due to the low proportion of the main chains that form the elastic network and the small molecular weight of the critical gel, it is not practical to use it as an elastomer.^53^ One solution involves the polymer fluid gel, synthesized by incorporating viscous fluid into dense elastic fibers, exhibits a high loss factor (tan > 0.5) across a broad frequency range (from 10^−2^ to 10^8^ Hz).^82^ On the other hand, by incorporating damping materials into other substrates, the damping properties of these materials can be significantly enhanced. For instance, introducing starch into hydrogels, which typically lack inherent damping capabilities, can transform them into composites with promising damping properties.^82,83^

**Fig. 5:**
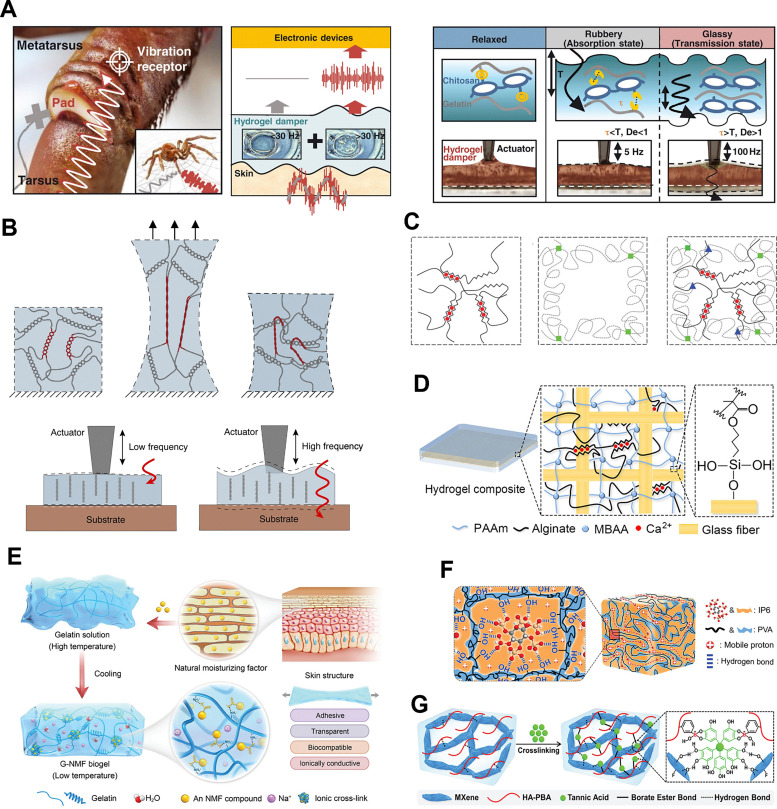
Design of damping materials based on hydrogels. (A) A selective frequency damper inspired by cuticular pads for bioelectronics with minimal dynamic noise interference. Reproduced with permission.^13^ Copyright 2022, American Association for the Advancement of Science. (B) A single-network κ-carrageenan hydrogel that achieves self-reinforcing and damping properties through the double-helix topological arrangement of its molecular structure. Reproduced with permission.^58^ Copyright 2023, Elsevier. (C) Illustration of ionic crosslinks in alginate (*via* Ca^2+^), covalent crosslinks in polyacrylamide (*via* MBAA), and an intertwined hybrid gel merging both networks with additional covalent linkages. Reproduced with permission.^84^ Copyright 2012, Springer Nature Limited. (D) Schematic of the hydrogel composite, consisting of a layer of glass fabric interpenetrated with the Ca-alginate/PAAm hydrogel. The PAAm chains are covalently anchored onto the surface of glass fibers *via* siloxane bonds. Reproduced with permission.^85^ Copyright 2022, American Chemical Society. (E) Schematic illustration of the biogel fabrication process. Reproduced with permission.^66,86^ Copyright 2024, Wiley-VCH GmbH. (F) Schematic illustration of the structure of the SF-supra-ICE. Reproduced with permission.^57,87^ Copyright 2023, Wiley-VCH GmbH. (G) Schematic illustration of the preparation of the adhesive, healable, and antibacterial MXene/HA-PBA/TA hydrogel. Reproduced with permission.^56^ Copyright 2024, Wiley-VCH GmbH.

A gelatin–chitosan-based hydrogel selective frequency damper,^13^ inspired by the corneous pad beneath a spider's vibration receptors, was reported recently to selectively filter out low-frequency mechanical noise (<30 Hz) while transmitting high-frequency signals (Fig. 5A). This frequency range is particularly well-suited for reducing noise in electrophysiological signal measurement. The hydrogel's damping mechanism arises from the separation of viscous bonds in the chitosan-based hydrogel and hydrophobic interactions in the gelatin-based hydrogel under external vibration stimuli, leading to the rearrangement of the polymer chains, allowing for selective frequency damping through the breaking and recovery of weak viscous bonds. Furthermore, a single-network kappa-carrageenan (KC) hydrogel reported recently (Fig. 5B) can achieve self-reinforcing and damping properties by utilizing its double-helix molecular structure, where cyclic stretching causes molecular entanglement that enhances tensile modulus, and high-frequency vibration triggers viscoelastic transitions for selective signal transmission and noise elimination.^58^ In addition, integrating multiple levels of crosslinking or fibrous reinforcement can further elevate the damping capacity of hydrogel systems. For example, hydrogels that simultaneously leverage ionic and covalent networks can exhibit substantial energy dissipation under mechanical disturbance, stemming from the partial rupture and subsequent re-formation of sacrificial bonds,^84^ as illustrated in Fig. 5C. The dual-network architectures lead to extraordinarily high stretchability while dissipating large amounts of energy through repetitive unzipping and rezipping of ionic crosslinks. Likewise, it has been demonstrated that reinforcing a tough hydrogel matrix with woven fibers and ensuring strong interfacial adhesion can impart both excellent load transfer and resilience to impact loads, underscoring the role of hierarchical structural design (Fig. 5D).^85^ Other examples include an all-natural biogel displaying tunable mechanical properties and stable biocompatibility for electrophysiological recording on both hairy plants and human tissue (Fig. 5E), underscoring the diverse potential of next-generation hydrogel adhesives to couple living organisms with functional devices.^66^ Moreover, a solvent-free supramolecular ion-conductive elastomer, as illustrated in Fig. 5F, leverages strong hydrogen-bonding interactions to support excellent ionic conductivity and tensile resilience, thereby forming “ionic tattoos” that adapt seamlessly to skin surfaces for on-skin bioelectronics.^57^ Meanwhile, a flexible conformally bioadhesive MXene hydrogel (Fig. 5G) for machine learning-facilitated human-interactive sensing integrates robust MXene nanosheets within a soft hydrogel framework to achieve strong bioadhesion and high signal fidelity, enabling real-time gesture recognition with minimal motion artifacts.^56^ Finely tuning polymer chemistry, crosslinking types, and structural assemblies can create hydrogel-based materials with tunable damping profiles capable of addressing vibration management challenges across a wide frequency range.

### 3.3. Acoustic metamaterials

Acoustic metamaterials typically employ intricately designed internal architectures, such as periodic arrays of resonator elements or labyrinthine chambers, to manipulate the propagation of mechanical waves.^15,51,88^ By guiding these waves along convoluted pathways, part of the energy becomes reflected or dissipated, allowing the structure to serve as a highly selective mechanical filter. Through meticulous tuning of parameters, such as the resonator size, arrangement, and internal porosity, one can precisely target and attenuate vibrations across specific frequency bands, effectively creating a band-stop or band-pass filtering effect.

Moreover, the ability to fabricate metamaterials from soft or flexible polymers, like 3D printing technology, broadens their applicability, making them suitable for contact with delicate, curved, or dynamically changing surfaces.^89,90^ For example, these metamaterials can be intergraded as intermediate layers or encapsulating shells in bioelectronics, leveraging their capacity to shield sensitive transducers and circuitry from mechanical noise. Despite the relative novelty of acoustic metamaterial research, recent progress underscores their immense promise in damping applications.

Other damping mechanisms also include a novel layered material strategy to mitigate motion artifacts in electrochemical signals.^91^ This approach involves three coordinated strain-energy dissipation mechanisms: (i) channel cracking in a brittle interfacial film, (ii) strain isolation of out-of-plane conductive pathways within the ACF layer, and (iii) reorganization of the in-plane silver nanowire. In particular, interface channel cracks act as a route for relieving tensile strain energy, allowing each fractured segment to experience minimal strain while still retaining electrical connectivity. As a result, the electrode maintains its overall active surface area even under mechanical deformation and reduces the motion-artifacts in electrochemical signals.

### 3.4. Summary

This section outlines strategies for engineering damping materials to minimize motion artifacts in the 0.01–50 Hz range typical of everyday activities. Table 2 offers a comparison of the SDMs covered in this section, illustrating how different chemistries and architectures can achieve the common goal of concentrating energy dissipation within a motion-artifact band while leaving the signal band unaffected. Commercial shear-thickening pads such as D3O® and DEFLEXION™ achieve impact absorption through particle-induced dilatancy or lattice buckling, providing solvent-free durability.^60^ However, their relatively high moduli and broadband loss factor impede efficient transmission of high frequency biosignals. By contrast, the ion-elastomers^57^ and pressure-sensitive adhesives^80^ exploit ion–dipole interactions or reversible hydrogen bonding to create adjustable high-tan *δ* band, and their solvent-free nature confers excellent long-term stability. Certain formulations, such as PTFEA-*co*-PFOEA,^52^ combine tan *δ* values above 1.0 across the motion-artifact band with negligible damping above 80 Hz, delivering excellent frequency selectivity. Hydrogel-based SDMs, including gelatin–chitosan^13^ and κ-carrageenan^58^ systems, offer the highest low-frequency tan *δ* values while maintaining low loss factors at higher frequencies, yet their lifetime is curtailed by water evaporation unless humectants or encapsulation layers are added. Finally, architected acoustic metamaterials convert vibration to heat through resonant scattering, furnishing highly adjustable dissipating frequency bands at the expense of thickness and stretchability.^15^ In broad terms, elastomers excel in long-term wearability, hydrogels in peak damping efficiency, and metamaterials in bandwidth customization, enabling researchers to align a given SDM with the mechanical and operational constraints of a specific soft-bioelectronic platform.

**Table 2:** Properties comparison of selective-damping materials

Ref.	Materials	Mechanism	tan *δ*_max_	*f*_damp_ band	tan *δ* at 100 Hz	Rebound resilience	Hydration stability	Applications
60	D3O	Proprietary polyurethane with shear-thickening dilatant particles	High	—	—	Low	Solvent-free	Electronic protection
60	DEFLEXION	Silicone elastomer with embedded hexagonal energy-absorbing cells	Medium	—	—	Low	Solvent-free	Clothes and shoes
79	Nematic liquid-crystal elastomer	LC side-chains, dynamic re-orientation	≈1.0	0.1–20 kHz	—	Low	Solvent-free	Low-frequency damping and sonic sealing
61	[EMI][TFSI]/P(VDF-HFP) ion-gel elastomer	Ion–dipole clusters, H-bonding	—	—	—	Medium	Solvent-free	Electronic skins
80	MEA/ATU pressure-sensitive adhesive	Reversible H-bond PSA	≈1.0	0–100 Hz	>0.5	—	Solvent-free	Adhesive
52	PTFEA-*co*-PFOEA + LiTFSI ion-elastomer	Phase-separated fluorinated blocks	>1.0	0.1–50 Hz	<0.05	Low	Solvent-free	Ionic skin for soft electronics and robotics
57,87	Supramolecular ion-conductive elastomer	H-bond quadruplexes in dry ionomer	>1.0	<100 Hz	Low	—	Solvent-free	ECG, EMG sensors
13	Gelatin–chitosan spider-pad hydrogel	Dual viscous bonds	>1.0	<30 Hz	<0.05	Low	Water loss with time	Electrophysiological sensors
58	κ-Carrageenan single-network hydrogel	Double-helix entanglement	>1.0	0.1–56 Hz	<0.05	Low	Water loss with time	Bioactuators
84	Ionic + covalent dual-network hydrogel	Sacrificial ionic links	<0.5	0.01–10 Hz	—	Medium	Water loss with time	Structure support
85	Woven-fiber–reinforced tough hydrogel	Woven fiber + matrix	—	—	—	Medium	Water loss with time	Impact protection
66,86	All-natural biogel	Protein–polysaccharide H-bond network	>1.0	<100 Hz	Low	Low	Limit water loss in 7 days	Electrophysiological sensors for plant/human
56 and 92	MXene/HA-PBA/TA bioadhesive hydrogel	Catechol cross-links, MXene	≈0.4	1–10 Hz	—	—	Water loss with time	Electrical skin, bioadhesive
93	Soft labyrinth acoustic metamaterial	PDMS matrix, 3-D resonant cavities	0.1	700–1000 Hz, adjustable	—	—	Solvent-free	Sound insulation

In summary, selective damping materials offer an attractive platform for fabricating long-term soft bioelectronics, owing to their adjustable operating frequency range and superior damping performance.

## 4. Applications of damping-material-based bioelectronics

### 4.1. Biosensing with mitigated motion artifacts

Soft bioelectronics represent a transformative advance in future medical technology, enabling real-time health monitoring and personalized therapy directly from the body's surface, thereby bridging the gap between clinical care and daily life health management.^25,94–96^ Specifically, they are capable of monitoring a variety of vital signals, such as electrophysiological signals,^94,97,98^ electrochemical signals,^1,99,100^ and physical signals.^95,101,102^ The causes of motion artifacts in biosensing electrodes vary significantly, depending on the type of signal that is being measured, therefore reducing motion artifacts across all signal types presents a substantial challenge, especially for multimodal soft bioelectronics. Each signal type responds differently to mechanical movements, making it difficult to develop a universal solution. For electrophysiological sensors, the device collects electrical signals generated by ion potential differences across cell membranes, which are then conducted through electrodes and converted into measurable voltage changes.^103–105^ Motion artifacts in electrophysiological signals caused by mechanical vibrations mainly stem from two factors: first, when the body moves, the contact state between the skin and the electrode changes, leading to fluctuations in interface impedance, which results in voltage variations that resemble biological signals; second, deformation of the electrode and its interconnects under dynamic strain generates additional strain-induced potentials, which are superimposed on the true physiological waveform, further degrading signal fidelity.^106,107^ Unlike electrophysiological signals, the impact of mechanical vibration on electrochemical sensors primarily affects the liquid–electrode interface.^75,108^ First, mechanical vibration increases the shear flow rate at the liquid interface, leading to fluctuations in the analyte concentration gradient. Second, vibration-induced disturbances at the liquid–electrode interface disrupt the capacitance between the electrode and electrolyte, generating noise in the sensor signal. Additionally, due to the fluid nature of the liquid, the position of the electrode interface may shift during vibration, resulting in artifacts in the sensor signal and making it challenging to accurately reflect the true analyte concentration.^36,109^ When the user is moving, shear forces, friction, and vibrations generated on the skin interface can disturb the fluid layer or the membrane medium, making it challenging to maintain a constant detection environment. For example, microfluidic flow rates, ion concentration gradients, or temperature distribution may all be altered by slight slippage or structural deformation. The incorporation of damping materials offers a novel approach to mitigating this issue. By introducing conductive SDM between the electrode and the skin, this impact can be effectively reduced, thereby improving signal stability and accuracy.

**Fig. 6:**
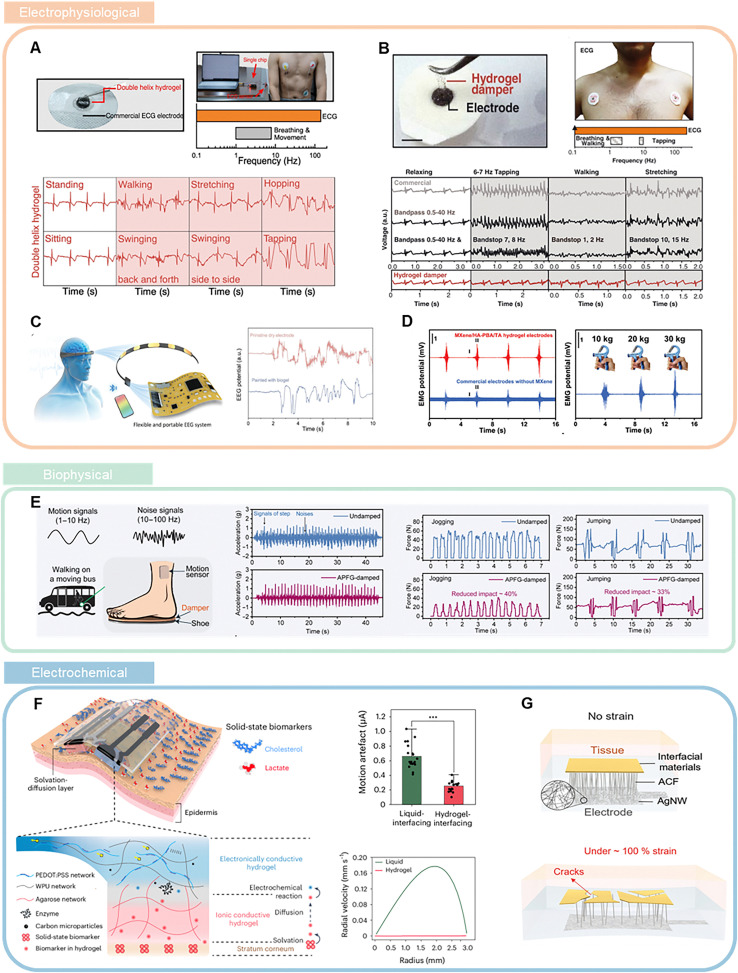
Biosensing applications of damping materials. (A) Example of bioelectronics using the single-network κ-carrageenan hydrogel electrodes for electrocardiogram (ECG) signal measurement, comparing bioelectronics and ECG detection on skin with hydrogel and commercial 3M electrodes. Reproduced with permission.^58^ Copyright 2023, Elsevier. (B) Demonstration of dynamic noise-damping using the selective frequency damper inspired by cuticular pads for high signal-to-noise ratio (SNR) detection of biophysiological signals. Reproduced with permission.^13^ Copyright 2022, American Association for the Advancement of Science. (C) Schematic illustration of the portable EEG acquisition head ring for wireless transmission and the EEG artifact signals with blinking collected by electrodes painted with and without GP biogel. Reproduced with permission.^66^ Copyright 2024, Wiley-VCH GmbH. (D) EMG signals measured by the MXene/HA-PBA/TA hydrogel electrode and commercial hydrogel electrode without MXene and the EMG signals measured while increasing gripper force. Reproduced with permission.^56^ Copyright 2024, Wiley-VCH GmbH. (E) Experimental setup for human motion sensing in noisy environments, illustrating motion signals (1 to 10 Hz) and noise signals (10 to 100 Hz) during walking on a bus, and comparison of the signal between undamped and damped motion sensing, force signals recorded during jogging, and force signals recorded during vertical jumping. Reproduced with permission.^55^ Copyright 2025, The American Association for the Advancement of Science. (F) Schematic illustration of the SEB sensor on the skin and a cross-sectional view of the SEB sensor, along with a comparison of motion artifact magnitude between the SEB sensor and traditional liquid electrochemical sensors. Reproduced with permission.^75^ Copyright 2024, Spring Nature. (G) Illustration of the soft strain-insensitive bioelectrode as a bioelectronics-tissue interface under strain. Reproduced with permission.^91^ Copyright © 2022, The American Association for the Advancement of Science.

Due to the double-helix molecular structure of single-network κ-carrageenan hydrogel,^58^ which effectively eliminates dynamic noise below 56 Hz, it holds great potential for mitigating motion artifacts in electrophysiological sensing applications, with its ability to absorb noise in electrocardiogram (ECG) signals already validated, as shown in Fig. 6A. Notably, measuring electrophysiological signals requires a stable conductive path between the skin and the electrode, necessitating that the SDM exhibits reliable conductivity. Since this hydrogel is inherently non-conductive, conductive materials like carbon nanotubes must be incorporated to achieve high conductivity. However, this approach may compromise the material's original properties. Additionally, inspired by the spider's keratinous pads, the gelatin–chitosan hydrogel damper selectively filters out low-frequency mechanical noise (<30 Hz) while transmitting high-frequency electrophysiological signals, enabling the acquisition of high-fidelity ECG signals across various movement states,^13^ as shown in Fig. 6B. Introducing damping materials has likewise proven highly effective for mitigating motion artifacts in other types of electrophysiological signals. For example, incorporating the newly developed moisturizing biogel into EEG acquisition equipment significantly diminishes the blinking-related artifacts often observed in EEG recordings, thereby improving signal clarity^66^ (Fig. 6C). In addition, MXene/HA-PBA/TA hydrogel electrodes have demonstrated the capability to reduce motion artifacts for EMG measurements, ensuring more stable and accurate muscle activity readings,^56^ as illustrated in Fig. 6D.

Biophysical signals, including temperature, pressure, and acceleration, provide critical information about the body's physical state and its interaction with the environment. These signals are also susceptible to strain-induced interference, resulting in motion artifacts. Incorporating SDMs into biophysical sensors can also help suppress the generation of such artifacts. For example, an armored polymer–fluid gel has recently been developed, which further demonstrate effective noise suppression across multiple types of biosignals (Fig. 6E).^55^

For electrochemical signals, a recent report introduced an electrochemical sensing system based on stretchable ionic–electronic bilayer hydrogels, which the solid hydrogel electrolyte can effectively reduce the motion artifacts of electrochemical signals caused by mechanical vibration,^75^ as illustrated in Fig. 6F. In addition, a novel layered composite design has been proposed to effectively dissipate mechanical energy in electrochemical electrodes^91^ (Fig. 6G). Although research on eliminating motion artifacts in electrochemical sensors is currently limited, enhancing sensor signal stability through the use of damping materials has shown considerable potential for future applications.

Overall, the integration of advanced damping materials in skin-interfaced soft bioelectronics presents a promising pathway to mitigate motion artifacts across multiple biosensing modalities. Continued development in this area could significantly enhance the reliability and accuracy of wearable health monitoring devices, particularly in dynamic, real-world environments.

### 4.2. Shock absorption

Another critical challenge for skin-interfaced soft bioelectronics is the inherent fragility of some crucial integrated components, such as the electronic components utilized for data processing, wireless data transmission, and powering. As the next generation of soft bioelectronics moves toward miniaturization and imperceptibility to enhance user comfort for long-term wear,^2,3,110,111^ the complexity and delicacy of integrated circuit designs increase. Achieving this miniaturization requires precise and densely packed electronic components, which are vulnerable to mechanical vibrations or external impacts. Such disturbances can damage the components, leading to signal artifacts or even complete signal loss.^112^ Moreover, some electrode materials typically used in soft bioelectronics, such as silicon,^113^ gold,^114^ and laser-induced graphene (LIG),^115^ are inherently brittle. This brittleness further exposes them to damage when subjected to vibrations and impacts, which degrades signal quality and compromises device reliability during extended use.

**Fig. 7:**
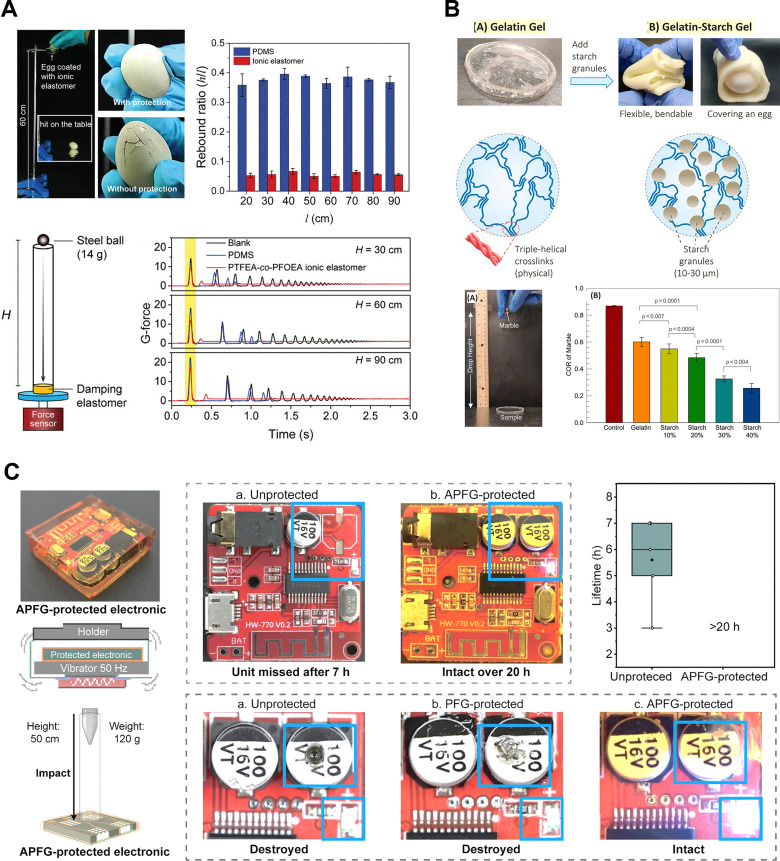
Impact-absorption applications of damping materials. (A) Photos of quail eggs wrapped with and without ionic elastomer after a 60 cm fall, and rebound ratios of PDMS and ionic elastomer are measured against falling height, and an experimental setup illustrates shock-controlling performance, with time-resolved G-force attenuation comparing a blank sample, PDMS, and ionic elastomer under different drop heights. Reproduced with permission.^52^ Copyright 2023, Wiley-VCH GmbH. (B) Comparison of the structures and impact performance (coefficient of restitution) of gelatin-based gels, both clear and turbid with added starch, showing their flexibility and ability to protect fragile objects, with results analyzed through rebound tests and statistical significance. Reproduced with permission.^65^ Copyright 2022, American Chemical Society. (C) Photograph and schematic of an electric board enclosed within APFG showing the vibrational damping setup, along with comparative images of boards after prolonged mechanical impact under unprotected and APFG-protected conditions. Reproduced with permission.^55^ Copyright 2025, The American Association for the Advancement of Science.

SDMs offer a practical method of protecting brittle electrodes and delicate micro-electronics by dissipating incoming mechanical energy before it reaches the device.^52,55^ SDM can absorb mechanical energy and reduce the effect of external vibrations and shocks, ensuring stable device operation even under dynamic conditions and during long-term use. To be effective in skin-interfaced bioelectronics, the ideal damping layer should be thin, highly compliant, and tough, so that it conforms to intricate component topographies without tearing or peeling.^4^ The protective power of SDMs can be illustrated by a simple experiment: experiments have shown that eggs wrapped in a thin layer of ion-elastomer, based on sticky fluorinated polymers,^52^ can withstand a drop from 60 cm without breaking (Fig. 7A). This demonstrates the potential of ion-elastomers to effectively absorb shocks and dissipate energy, highlighting their application in bioelectronics for protecting delicate components.

Effective shock dampers should not only absorb the initial blow but also minimize energy return, ensuring that sensitive electronics experience just one, highly attenuated mechanical impulse. Therefore, in addition to shock absorption, another key property of damping materials is rebound resilience, which refers to a material's tendency to rebound after absorbing impact. The rebound resilience of a damping material critically determines how much residual stress is transmitted back into a soft bioelectronic system once the primary shock has been absorbed.^52,116,117^ A low rebound ratio signifies that the material converts most of the impact energy into heat and thus prevents a bounce-back pulse from propagating into fragile interconnects. Materials with high rebound resilience, like polydimethylsiloxane (PDMS), dissipate less energy and are more prone to damage from sustained vibrations. In contrast, materials that exhibit minimal rebound are better suited for damping purposes. For instance, a comparison between the rebound resilience of PDMS and a PTFEA-*co*-PFOEA ion elastomer (measured as the ratio of rebound height to drop height) revealed that the ion elastomer has a significantly lower rebound ratio—approximately 0.05 compared to around 0.37 for PDMS.^118^ This illustrates the superior damping ability of ion-elastomers and their potential to safeguard fragile bioelectronic devices from external impacts. Similarly, recently developed hydrogel systems that incorporate starch particles have demonstrated effective shock absorption for fragile objects,^65^ with the impact-absorbing properties being tunable by varying the starch content (Fig. 7B). This flexibility in design highlights the potential for such hydrogel-based materials to be applied in bioelectronics as protective and energy-absorbing layers. By adjusting material compositions, researchers can optimize these systems to provide enhanced mechanical protection while maintaining the flexibility and conformability needed for skin-interfaced bioelectronics. Additionally, the armored polymer–fluid gels damper demonstrates excellent damping properties under both prolonged vibrations and sudden impacts, thereby providing protection for devices embedded within the host material,^55^ as illustrated in Fig. 7C.

### 4.3. Clinical potential of SDM-integrated bioelectronics

SDM-integrated wearable bioelectronics have shown outstanding ability in laboratory studies to suppress motion artifacts and protect fragile electronic components, highlighting their considerable clinical potential. Recent progress is now moving these SDM-based devices beyond proof-of-concept prototypes toward clinical trials. For example, the Janus adhesive hydrogel (JAH) is capable of selective respiration noise damping by modulating energy dissipation.^14^ JAH has been utilized for clinical trials, including high-sensitivity non-invasive diagnosis of otitis media and polysomnographic monitoring of obstructive sleep apnea (OSA). For otitis media diagnosis, compared with traditional invasive methods (such as metal probes penetrating the subcutaneous to the skull), non-invasive skin interface electrodes developed by JAH (directly adhered to the scalp without piercing the tissue) can achieve higher detection sensitivity. Furthermore, for clinical OSA assessment, electrophysiological electrodes based on JAH can obtain polysomnography (PSG) with higher accuracy than commercial electrodes, and have significant dynamic noise attenuation in the range of 0.1–1 Hz. These are attributed to the excellent damping properties of the hydrogel damping material. On the other hand, a MEMS pressure array laminated onto a viscoelastic silicone substrate suppresses skin-stretch–induced artifacts in arterial pulse–wave recordings, thereby enabling precise heart rate monitoring and accurate arrhythmia detection.^119^ This device has already been clinically deployed in 25 cardiology patients (seven with atrial fibrillation and 18 recovering from cardiac surgery) and has collected high-fidelity arterial pulse signals throughout monitoring. In addition, several flexible devices that incorporate damping materials have already reached the market. A representative example is the 3M conductive adhesive electrode, whose integrated viscoelastic gel enhances conformal skin contact while simultaneously acting as a cushioning layer to suppress external mechanical noise.^13^ Collectively, these early clinical trials signal a broad clinical horizon for SDM-integrated bioelectronics. By combining high damping efficiency with soft, skin-conformal form factors, SDMs elevate signal fidelity across diverse modalities while eliminating the discomfort and infection risks inherent to invasive probes. Such improvements enable longer, more reliable monitoring sessions in both hospital and home settings, supporting data-rich precision diagnostics and continuous disease management. Although long-term durability and large-scale validation remain to be fully established, the consistency of clinical benefits observed to date underscores the large potential of SDM-integrated bioelectronics for next-generation clinical care.

In summary, the application of SDM in soft bioelectronics offers a promising solution to the challenge of protecting fragile electronic components and brittle electrode materials. Through careful material selection and engineering, SDM can ensure device longevity, stability, and high performance, even in environments prone to mechanical stress and vibration.

## 5. Challenges and conclusion

Due to their exceptional energy dissipation and selective damping capabilities, SDM have enabled various advancements in skin-interfaced soft bioelectronics, particularly in applications like biosensing and shock protection. These developments aim to achieve stable, long-term health monitoring free from motion artifacts, which can severely compromise data quality. The selective damping characteristics of SDM are especially appealing because they mitigate motion-induced noise without requiring additional mechanical components or sensors for noise reduction. Moreover, SDM offer precise targeting of specific noise frequencies and thereby enhancing the accuracy of biosignal monitoring. Although current SDM-based soft bioelectronics applications remain limited to proof-of-concept demonstrations, this area holds immense potential. The inherent ability of SDM to dissipate energy makes them ideal candidates for wearable devices, as they can maintain signal integrity without external systems for artifact reduction.

However, significant challenges remain, especially when translating these innovations from laboratory settings to practical, real-world applications. Firstly, biocompatibility constitutes an important consideration for all selective-damping materials intended for extended skin contact. Most SDM formulations reported to date are based on polymers and fillers, such as PDMS, medical polyurethanes, silicone pressure-sensitive adhesives, κ-carrageenan, gelatin, chitosan, and FDA-listed ionic liquids (*e.g.*, EMI-TFSI), that have previously shown acceptable short-term biocompatibility.^58,80,94–96,120,121^ Several SDMs have also undergone standard biocompatibility and cytotoxicity assessments: for example, Janus adhesive hydrogel (JAH) maintained 94.27% cell viability after 24 h exposure and produced no observable inflammatory response in skin tissue;^55^ the solvent-free supramolecular ion-conductive elastomer (SF-supra-ICE) produced no irritation after a 3-day dorsal application in mice and showed no systemic toxicity in a 42-day gavage study, indicating good biocompatibility;^57^ the all-natural GP biogel retained 96.4% viability of MC3T3-E1 cells after 48 h incubation and elicited no adverse reaction during a 48 h human wear test, likewise demonstrating low cytotoxicity;^66^ and extracts of the MXene/HA-PBA/TA hydrogel supported normal proliferation of L929 fibroblasts for up to 72 h, confirming reliable biocompatibility.^56^ However, some SDMs have not undergone complete biocompatibility and cytotoxicity testing, posing potential application risks. For instance, hydrogel-based SDMs are built from biopolymers that are generally regarded as safe, yet they gradually dehydrate and their networks break down over time, and the resulting degradation products and their long-term effects during continuous wear remain largely uncharacterized.^122,123^ Furthermore, elastomeric SDMs may release trace monomers, photo-initiators or ionic additives during prolonged use, and the dermatological impact of such leachables is still under investigation. For architected acoustic metamaterials, PDMS foams and resonant polyurethane lattices are themselves biologically inert and essentially non-degradable over the product lifetime, yet no published studies have evaluated its biocompatibility. In short, while initial biocompatibility data for SDMs are promising, comprehensive biocompatibility evaluations are still lacking, and closing this gap will be essential before SDM-integrated bioelectronics can transition from laboratory prototypes to routine clinical wearables.

Another primary material-related issues lies in the long-term stability of SDMs. Elastomer-based SDMs and acoustic metamaterials usually have long-term stability due to their solvent-free characteristics, while hydrogels tend to dry out and degrade over time, and high temperatures and low humidity may accelerate dehydration.^124–126^ This structure change compromises their functionality, making them less suitable for applications that require sustained performance over extended periods. Furthermore, achieving stable, conformal contact between the device and skin is essential for high-fidelity signal acquisition in soft bioelectronics. Poor contact increases motion-induced artifacts, leading to elevated noise levels and degraded signal quality.^4^ To mitigate this, devices must adhere closely to the skin surface, which poses a challenge for many SDM materials. Although SDM exhibit excellent damping properties, their effectiveness often requires relatively thick layers, which can hinder practicality in soft bioelectronics where thin, lightweight materials are preferred. Additionally, many SDM materials exhibit low adhesion to skin, further complicating their use in long-term wearable applications. One approach to overcoming this challenge has been to combine SDM with commercially available adhesives. However, this introduces complexity in device fabrication and may lead to material incompatibility, further complicating the integration of SDM into practical bioelectronics. On the other hand, from a fundamental perspective, current research on damping materials still focuses primarily on mitigating motion artifacts in electrophysiological signals. However, effective healthcare monitoring typically demands measurement of multiple physiological signals, such as electrochemical signals, and motion artifacts in these other types of signals remain insufficiently explored. Moreover, most skin-interfaced bioelectronics solutions to date revolve around device level implementations, with relatively few attempts to build fully integrated systems at the system level.

Another practical challenge in translating SDM research into products is whether micro-architecture systems (specifically phase-separated ion-elastomers and acoustic metamaterials) can be manufactured at commercial scale. Phase-separated ion-elastomers appear well positioned for volume production: the material is formed by simply photopolymerizing a cast precursor, a process already compatible with roll-to-roll coating and slot-die extrusion. Their intrinsic self-healing also means off-cuts and post-consumer scrap can be ground, remixed with monomer, and re-cured, offering a straightforward recycling pathway. Acoustic metamaterials are more challenging because their performance depends on precisely tailored internal cavities; scaling them therefore requires either high-resolution additive manufacturing or complex mold inserts, both of which raise cost and throughput concerns.

To broaden the application of SDM in soft bioelectronics, future research must address these challenges by developing materials that offer reliable performance along with the necessary comfort, stability, and environmental safety for long-term use. For instance, the dehydration issue in hydrogel-based SDM could be mitigated by incorporating anti-drying agents like glycerol,^94,127,128^ which would help maintain their structural integrity over time. Similarly, the widespread problem of low adhesion could be tackled through material innovation and structural engineering. One promising solution involves the development of SDM composites that integrate adhesive layers. These composite materials would combine the damping properties of SDM with the adhesive strength required for stable skin contact, thereby improving both performance and comfort in wearable devices. In addition, interdisciplinary research in areas such as chemistry, electric engineering, and medicine is also crucial to deepen the understanding of multi-signal motion artifacts, thereby further propelling the development of system-level, artifact-free skin-interfaced bioelectronics.

In conclusion, given their outstanding energy-dissipation capabilities, SDM have the potential to revolutionize long-term skin-interfaced bioelectronics, paving the way for more reliable, comfortable, and environmentally safe wearable technologies. By addressing current material limitations, future developments in SDM could lead to significant advancements of soft bioelectronics in precision healthcare, human-machine interfaces, and other applications.

## 6. Methodology

An exhaustive electronic search was performed in Web of Science, Scopus, PubMed, IEEE Xplore, and ClinicalTrials.gov. To focus on damping materials and the most recent advances, the core query for soft or skin-interfaced bioelectronics was combined with the modifiers damping, viscoelastic, hydrogel, elastomer, or acoustic metamaterial. Reference lists of all retrieved articles were hand-screened to capture additional studies.

Papers were included if they (i) reported original *in vitro*, *in vivo*, or human experimentation on skin-mounted soft bioelectronics that integrate a damping or energy-dissipative layer/material; (ii) provided quantitative metrics such as motion-artifact attenuation, signal-to-noise-ratio improvement, shock-absorption capacity, or device reliability; and (iii) were peer-reviewed, English-language articles published in reputable journals.

Each eligible study was then evaluated with a modified Joanna Briggs Institute (JBI) checklist encompassing five domains: (1) design transparency (clear description of materials, fabrication, and test set-up); (2) sample adequacy (sufficient repeat numbers or independent batches); (3) appropriate controls or baseline comparisons; (4) completeness of quantitative reporting (means ± SD/SE, statistical tests); (5) reproducibility indicators (independent replication or multi-batch validation). Studies fulfilling ≥4 of these criteria were classified as low risk of bias, those meeting 2–3 as moderate risk, and ≤1 as high risk. High-risk articles will not be cited.

## Author contributions

Zehua Chen: writing – review & editing, writing – original draft, visualization, conceptualization. Feng Zhang: writing – review & editing, writing – original draft, conceptualization. Xiaoyan Qian: visualization, validation. Ganggang Zhao: visualization, validation. Zheng Yan: writing – review & editing, writing – original draft, supervision, project administration, funding acquisition, conceptualization.

## Conflicts of interest

There are no conflicts of interest to declare

## Data Availability

No primary research results, software or code have been included and no new data were generated or analyzed as part of this review.
